# Does professionalism change with different sociodemographic variables? A survey of Arab medical residents

**DOI:** 10.1080/07853890.2022.2105390

**Published:** 2022-08-20

**Authors:** Eiad Alfaris, Farhana Irfan, Fahad D. Alosaimi, Shaik Shaffi Ahamed, Gominda Ponnamperuma, Abdullah M. A. Ahmed, Hisham Almousa, Naif Almutairi, Tamim Alwehaibi, Mohammad AlQuaeefli, Faisal AlFwzan, Tareq Alomem, Mohamed M. Al-Eraky

**Affiliations:** a Department of Family and Community Medicine, College of Medicine, King Saud University Chair for Medical Education Research and Development, King Saud University, Riyadh, Saudi Arabia; b Department of Psychiatry, College of Medicine, King Saud University, Riyadh, Saudi Arabia; c Department of Medical Education, Faculty of Medicine, University of Colombo, Colombo, Sri Lanka; d College of Medicine, King Saud University, Riyadh, Saudi Arabia; e Department of Medical Education, Imam Abdulrahman Bin Faisal University, Dammam, Saudi Arabia

**Keywords:** Professionalism, residents, professional ethics, medical education/postgraduate, medical education/graduate

## Abstract

**Background:**

Medical professionalism reflects the commitment of physicians to their patients, society, themselves, and the profession. The study examined residents’ attitudes towards professionalism and how these attitudes vary among the different demographic groups, namely gender, specialty, and year of residency.

**Methods:**

A proportionate random sampling strategy was used to select the study sample. Medical residents from six specialties at a large tertiary care teaching facility were invited to participate in an online survey. The survey used the modified Learners Attitude of Medical Professionalism Scale (LAMPS), which consists of five domains: respect, excellence, altruism, duty/accountability, and integrity. Chi-square, Student t-test, one-way ANOVA, factorial ANOVA, and post hoc analysis were used to examine the attitudinal differences towards professionalism among the different demographic factors.

**Results:**

The overall response rate was 82.7%. Overall, the residents’ self-reported attitudes towards professionalism was positive. The highest score was for the “respect” domain (4.61), and the lowest was for “altruism” (3.67). No significant association was found between the mean scores and the three studied variables, namely, gender, specialty (surgical/nonsurgical), and level (senior/junior).

**Conclusions:**

No significant differences were observed in the overall attitude towards professionalism among the residents regarding their year of residency, gender, and specialty. The low altruism score and absence of improvement of the total score regarding the residents’ increasing experience in the profession are concerns that need remedial action. Therefore, we suggest that future research look for possible explanations by using multi-institutional surveys that explore not only the residents’ attitudes, but also the trainers’ attitudes and practice, work situations, the hidden curriculum, and culture.

**Key messages**
- Attitudes towards professionalism among different demographic groups of residents do not show similar variations as has been reported in the literature, albeit in different sociocultural contexts.
- The low altruism score and absence of improvement of the total score as the residents gained more experience in the profession are concerns that need remedial action.
- A longitudinal study involving more than one institution for both residents and their faculty members to compare faculty scores with those of residents, while controlling for specialty and gender, may help elucidate the factors affecting attitudes towards professionalism and suggest possible means of addressing unfavourable attitudes.

## Introduction

Medical professionalism is a moral phenomenon and can be defined as the maintenance of certain desired behaviours and attitudes by medical professionals while serving their patients and society [1–3]. In this sense, professionalism is the basis of medicine’s contract with society and serves as a cornerstone in the physician–patient relationship. Nowadays, with globalization, competency related to professionalism, namely the physicians’ capacity to deal with cultural diversity, has gained increasing attention within the global healthcare context [4].

The importance of professionalism has been well documented in the literature [5–8], and for patients, it is considered the force that drives doctors to do right whenever they treat patients. The core values that medical professionals embrace when serving patients include trust, respect, honesty, the ethical use of resources, and social justice [9].

In the early 1990s, professionalism emerged as an important educational topic for health workers. There were fresh calls for a renewed sense of professionalism calling for a greater focus on professionalism in the education of both residency and medical school curricula [10]. This was mostly the result of societal shifts of expectations and the increasing concerns about ethics and professional misconduct occurring across the globe [1,11]. According to the American Board of Internal Medicine (ABIM), professionalism includes altruism, accountability, excellence, duty, service, honour, integrity, and respect for others; the ABIM views professional responsibilities as a commitment to maintaining professional competence [9].

For physicians in training, many factors can influence their professional behaviours, including demographics (gender, level of training [senior versus junior]), philosophy of the curriculum (i.e. a curriculum designed to be more patient-centered as opposed to being theory oriented), methods of teaching, education about professionalism, and institutional values [12,13]. Many earlier reports have shown that students’ gender plays an influential role in shaping attitudes towards medical professionalism. Female students have been found to be more likely to label behaviours as unprofessional [14,15]. In addition, a Canadian study among medical students and residents showed females to be more likely to perceive improper actions as unprofessional and to demonstrate higher attitude scores than their male counterparts across almost all domains [16]. The same study and two others found that attitude scores declined as the students progressed through medical school [14,16,17]. In a study among residents from surgical and nonsurgical training programs, surgical residents rated themselves higher than nonsurgical residents in explaining medical treatment options to patients [18].

The literature on medical professionalism has explored the experience of medical students and, to a lesser extent, among residents (mainly in the Western world). Previous studies used paradigms that may not be well fitted to the cultural values of non-Western countries [19]. In addition, Ho et al. [20] concluded that Western frameworks classify physicians’ professional and personal lives, while many Asian countries have cultural/traditional influences towards balancing these roles. A study [21] on professionalism in the local context indicated that Western models may not be applicable to non-Western cultures because of many differences, such as personal faith. This is supported by a study on the expectations of professionalism by stakeholders’ from Taiwan and China outlining the effect of values, as one size does not fit all [19].

Lapses in professionalism do happen, so assessing professionalism is the way to identify, correct, and explore the possible causes for these lapses. As said by Stern, “If it can’t be measured, it can’t be improved,” and “They don’t respect what you expect; they respect what you inspect” [22].

Residency programs have struggled with how to operationalize the learning of professional behaviours. Despite endeavours to uniformly incorporate professional behaviour among trainees, professional behaviours differ among residents in different specialties.

In the study institute, King Saud University Medical City (KSUMC), we have observed some forms of subtle unprofessional behaviours, such as instances of unintended disrespect. These types of behaviour have been observed in the healthcare environment and include behaviours arising from students, residents, and other healthcare professionals. A literature search revealed that most studies have focused on students, with only a handful addressing professionalism attitudes in the context of residents in different specialties in the Arab region [23–26]. Either these studies concentrated on a single specialty or did not compare specialty, gender, or year of residency within the same study. This prompted us to assess professionalism. The following questions guided our study:

What are the attitudes of residents towards professionalism? How do these attitudes vary among the different demographic groups, namely gender, specialty, and year of residency?

## Objectives

1. To assess the attitudes towards the medical professionalism of residents within the specialties of family medicine, internal medicine, obstetrics and gynaecology, paediatrics, emergency medicine and general surgery (both when compared individually by specialty and when compared after reclassifying the participants into surgical and nonsurgical specialties).
2. To discover whether the demographic variables of gender, year of residency, and specialty would influence the individual’s attitude towards professionalism.

## Materials and methods

### Design

A cross-sectional study was performed using an online questionnaire. The CROSS reporting guidelines were used.

### Study setting

King Saud University Medical City (KSUMC) is a 1400-bed tertiary care medical teaching facility and one of the largest postgraduate training centres in the Gulf region. It offers numerous postgraduate training programs, including family medicine, internal medicine, obstetrics and gynaecology (Ob-Gyn), general surgery, paediatrics, anaesthesia, emergency medicine, ophthalmology, orthopaedics, radiology, ENT, and psychiatry. The duration of residency training varies from four to five years. In addition, KSUMC offers 28 fellowship programs.

The residency programs of King Saud University accept graduates from all Saudi medical schools as residents. Although the medical schools follow the same Saudi learning outcomes (SauidMeds), in real life, there are major differences in both the content and teaching strategies used in these schools.

The Saudi Commission for Health Specialties (SCFHSs) regulates all postgraduate medical and other health training programs and accreditation in Saudi Arabia. It has adopted the Canadian Medical Education Directives for Specialists (Can-MEDS) competency framework for developing curricula for postgraduate training programs [27]. The KSU institutional framework puts greater emphasis on the theoretical knowledge base of professionalism in the initial years of undergraduate studies and its application to a clinical environment. At the postgraduate level, the curriculum is exemplified by a strategy of presenting professional dilemmas or challenging situations and assessing residents’ actions and decisions. To foster professionalism, the centre of postgraduate studies at KSUMC has applied several teaching and assessment methods to maintain the standard among all specialties. The Department of Medical Education runs professional development workshops for residents and faculty. The SCFHS online modules and handbook on professionalism are sources to be used by all postgraduate training programs. The institution (KSUMC) has integrated this framework into the accreditation standards, specialty training, final in-training evaluations, and exam blueprints for program certification. The residents are monitored and assessed through formative and summative evaluation, end-of-year examinations, in-training evaluation report/observations, and 360 feedback, and final Saudi board examination [28]. However, it remains challenging for each program to incorporate professionalism teaching within day-to-day clinical work.

### Sample/participants

A stratified random sampling technique with the proportional allocation method was used to determine the number of residents based on gender, specialty, and training year. The inclusion criterion was being a medical resident of either gender, being in one of six specialties (family medicine, internal medicine, obstetrics & gynaecology, paediatrics, emergency medicine, or general surgery), at any point in residency, and being at the KSUMC. The exclusion criteria were residents from other specialties and those working outside KSUMC.

Raosoft^®^ software was used to calculate the required sample size . The sample size was estimated based on the standard deviation of the professionalism score (the study outcome variable). Because the maximum total score is 155 (no of items * maximum score), we used a range of the standard deviation of scores of professionalism (between 35 and 39), 5% margin of error (with a 95% confidence interval); the estimated sample size was found to be between 200 and 234. With an expected nonresponse of 40%, the estimated sample size was increased to 329. A stratified random sampling technique using the proportional allocation method was used to determine the number of residents from each of the three strata, that is, gender, specialty, and training year. Microsoft Excel was used to generate random numbers to select the study sample within each gender, specialty, and training year.

None of the returned questionnaires were excluded from analysis because there were no missing data.

## Ethics and consent to participation

We obtained the participants’ electronic consent and permission to be included in the study. A cover letter acc0mpanied the questionnaire, explaining the aims of the study, assuring confidentiality, and that the information gathered will be used exclusively for research purposes. The current study was approved by the Institutional Review Board of King Saud University (project No. E-19-4416).

### Conceptual framework

Ecological systems theory [29] was used in the current study to understand residents’ professionalism development over time and the effects of social ecological components on their development. This provided a method for interpreting professionalism with respect to the interaction between the residents and their social and cultural contexts as they progress through the four/five years of their programs. Professional development mapped over time (the chronosystem) is reflected in this study by the training years. The individual interactions (microsystems) among the different groups of trainees are represented by resident gender. The curriculum and institutional/care settings (mesosystems), community/wider local context (exosystems), and culture (macrosystems) are the other components applicable to the respective counterparts of the framework.

## Instrument

Over the past few decades, various instruments have been developed to assess medical professionalism. However, a literature search on measuring professionalism in an Arabian context found the LAMPS inventory as promising because it was designed to be a culturally specific inventory for Arabs and is intended to measure the attitudes of medical students and residents towards medical professionalism [12]. The LAMPS inventory consists of 28 items and is one of the first valid and reliable questionnaires that is comparable to the tools developed for the same purpose in both Western [13] and Eastern [6] contexts. The questionnaire has two sections: a short demographic questionnaire to collect information on the study variables, that is, age, gender, specialty, and residency year of the participants. We also used the modified Learners Attitude of Medical Professionalism Scale (LAMPS) inventory (Appendix Table A1).

**Table 1.** Physicians’ professionalism mean total score in relation to gender, year of residency, and specialty.

Study variable	No (%)	Mean score (SD)	*t-*Value/*F*-ratio	*p*-Value
**G**ender				
Male	131 (48.17%)	4.26 (0.32)	1.445	.150
Female	141 (51.83%)	4.20 (0.29)		
Year of residency*				
R1	72 (26.47%)	4.24 (0.29)	2.415	.049*
R2	67 (24.63%)	4.24 (0.31)		
R3	67 (24.63%)	4.14 (0.33)		
R4	58 (21.32%)	4.28 (0.26)		
R5	8 (2.94%)	4.40 (0.37)		
Specialty				
Internal medicine	84 (30.88%)	4.21 (0.28)	1.684	.139
Family medicine	53 (19.49%)	4.22 (0.33)		
Paediatrics	50 (18.38%)	4.20 (0.33)		
Emergency medicine	32 (11.76%)	4.18 (0.32)		
Obstetrics and gynaecology	28 (10.29%)	4.37 (0.25)		
General surgery	25 (9.19%)	4.27 (0.28)		

*Post hoc analysis showed that no significant difference between different years.

The modified LAMPS consists of 31 items because it has three added items to the original 28 items of the LAMPS, which is a validated tool with a reliability of 0.79 [30].

The items of the study instrument (modified LAMPS) were classified into the following five domains (subscales):

1. Respect, with six questions
2. Excellence/autonomy, with six questions
3. Altruism, with five questions
4. Duty/accountability, with seven questions
5. Honour/integrity, with seven questions

The three items added to the original 28 items related to lapses in academic integrity were extracted from the Dundee Polyprofessionalism Inventory I [31].

Two items related to the “honor/integrity” domain were “signs the attendance paper in scientific meetings on behalf of his/her colleagues or asks them to sign it in one’s own absence” and “takes the work or idea from a colleague and using it as one’s own without acknowledging its source.” The other item from the “respect” domain was “being late for scientific meetings with colleagues and medical staff, without an excuse.”

A 5-point Likert scale (ranging from strongly disagree to strongly agree) was used as the response format.

To assess content validity, five experts were consulted (two ethicists, a community medicine specialist, and two medical educationists) regarding the suitability of the three items to be added to the original LAMPS inventory. The modified LAMPS was pilot tested among 20 family medicine residents. After a tutorial on professionalism, the residents were asked to fill out a hard copy of the instrument in the presence of the first author. The items that were found to be difficult to comprehend were rewritten to avoid misinterpretation and facilitate understanding.

The participants taking part in the pilot test were similar to the sample population.

## Data collection

An online questionnaire was administered en bloc; no interviewers were required. The preparation process included contacting the director of postgraduate studies, who sent direct email to each selected participant.

The questionnaire was uploaded, and the link was emailed via Google forms to the residents, with a supporting letter from the Director of the Postgraduate Studies. We also attached a cover letter explaining the aims of the study, assuring participants of data confidentiality and anonymity, and appealing for their professionalism in diligently filling out the instrument. Participants taking the survey multiple times was prevented by requesting the residents’ identity number, here with a pledge for confidentiality given by the researchers. Informed consent to participate was obtained. The data collection process began in February 2020 and lasted for one month. Two reminder emails were sent to those residents who did not respond.

## Data analysis

The Statistical Package for the Social Sciences (SPSS, version 26, IBM Corp., Armonk, NY, USA) was used for data analysis. No missing data were encountered because the questionnaire was uploaded to Google forms, and the respondents could not proceed without filling out all the previous items.

### Potential outcomes

The main outcome is the quantification of attitudes towards professionalism among Saudi medical postgraduates. As a result, the current study can help identify the groups of postgraduate trainees who need more support to improve their attitudes towards professionalism.

For positively worded items that represent professional behaviours, we used a scale of 1 to 5 (1 = strongly disagree, 5 = strongly agree). For negatively worded items that represent unprofessional behaviours, the scale was reversed (1 = strongly agree, 5 = strongly disagree). The mean and standard deviation for each item, domain, and total score were reported, along with the reliability, using Cronbach’s alpha.

Analyses for the differences in the total modified LAMPS mean score and the domain mean scores within each of the three variables (gender, specialty, and year of residency) were conducted using either a Student’s *t*-test for variables with two categories or one-way ANOVA for variables having more than two categories. *Post hoc* analysis was done with Tukey’s multiple comparison test. Furthermore, three-way analysis of variance was carried out by reclassifying the specialties into surgical and nonsurgical and the residency years into junior (R1 & R2) and senior (R3, R4, & R5) and including gender as unchanged so as to observe the effect of two- and three-way main and interaction terms, respectively, on the modified LAMPS mean scores. The results were interpreted using a significance level of *p* < 0.05.

## Results

### Overall sample statistics

#### Response rate

The number of respondents who completed the survey were divided by the number of total assigned (272/329), and this was similarly done for other demographic characteristics such as specialty.

Among the 329 residents who were invited to participate, 272 (82.7%) agreed to participate. The highest response rate was from emergency medicine residents (88.9%), followed by family medicine (88.3%), internal medicine (85.7%), and obstetrics and gynaecology (73.7%). The baseline demographic characteristics of the residents are presented in Table 1. The proportion of the study sample is similar to the target population regarding gender and year of study.

The Cronbach’s alpha reliability coefficient for the 31 items was 0.814. The overall residents’ self-reported attitude towards professionalism was positive. The highest mean score was for the “respect” domain (4.61), followed by “excellence/autonomy” (4.37), “honor/integrity” (4.31), and “duty/accountability” (4.09). The lowest score was for “altruism” (3.67).

The mean item scores varied between 3.12 and 4.74 (Appendix Table A1). The highest item score (after reverse coding) was for “give priority to some patients based on social status or nationality” in the domain “respect to others.” The lowest score was for the item “calls the insurance company to follow up a valid patient claim” in the “duty/accountability” domain.

### Participants’ demographic characteristics versus total mean attitude scores towards professionalism

Out of the 272 respondents who filled out the LAMPS inventory, 141 (51.8%) were females (Table 1). Most residents were from internal medicine (30.9%), followed by family medicine (19.5%) and paediatrics (18.4%) (Table 1). The majority of residents were of Saudi origin and had been employed in training posts.

#### Variation of professionalism-related attitudes with resident gender

The residents’ gender did not produce a significant difference regarding the total score (Table 1). The exception was for “duty/accountability,” where male residents had a statistically significant higher total mean score (4.17) compared with their female counterparts (4.02) (*p* = .005) (Table 2).

**Table 2.** Mean scores for males and females in the professionalism domains.

	Respect	Excellence/autonomy	Altruism	Duty/accountability	Honour/integrity
Male	4.59	4.40	3.66	4.17	4.34
Female	4.625	4.33	3.675	4.02	4.29
*p*-Value	0.501	0.140	0.868	0.005	0.343

#### Variation of professionalism-related attitudes with residency year

Regarding the effect of residency year, the one-way ANOVA of the modified LAMPS total mean scores revealed a significant effect for the year of program (*F* = 2.415, *p* = .049) (Table 1). The effect of the year of training on professionalism indicated that the mean total scores were all different: fifth year (4.40 + 0.37) was greater than fourth year (4.28 + 0.26), and both were greater than the third and entry years (4.14 + 0.33; 4.24 + 0.29) (Table 3). However, *post hoc* analysis of the residency year mean total scores did not reveal a significant difference between specific years.

**Table 3.** Mean scores of the five professionalism domains in relation to year of residency (only the significant findings are shown).

Variable	Number	Mean	*t*-test/*F*-ratio	*p*-Value
**Respect**				
R1	72	4.65	4.731	.001
R2	67	4.64		
R3	67	4.43		
R4	58	4.71		
R5	8	4.62		
*Post hoc* analysis showed a significant difference between R3 and R1, R2, and R4.				
**Altruism**				
Year of residency				
R1	72	3.64	4.179	.003
R2	67	3.73		
R3	67	3.57		
R4	58	3.67		
R5	8	4.1		
Post hoc analysis showed a significant difference between R5 (highest) and R1, R3, and R4.				
Specialty				
**Duty/Accountability**				
Year of residency				
R1	72	4.18	3.505	.008
R2	67	4.03		
R3	67	3.97		
R4	58	4.15		
R5	8	4.39		
*Post hoc* analysis showed a significant difference between R3 (lowest mean) and R1.				

Table 3 shows the mean professionalism domain scores from entry to end of training (year 5).

Univariate tests on levels of residency year revealed significant differences among the mean scores in the domains of “respect” (*p* = .001), “altruism” (*p* = .003), and “duty/accountability” (*p* = .008) but not for the honour/integrity and excellence domains (Table 3).

#### Variation of professionalism-related attitudes with specialty

Analysis of the different specialties showed significant differences in the mean scores of the “altruism” domain (*p* = .005) and “honor/integrity” domain (*p* = .018) (Table 4).

**Table 4.** Mean scores of the five professionalism domains in relation to specialty (only the significant findings are shown).

Variable	Number	Mean	*t*-Value/*F*-ratio	*p*-Value
**Altruism**				
Family medicine	53	3.64	3.401	.005
Internal medicine	84	3.61		
General surgery	25	3.76		
Obstetrics & gynaecology	28	3.92		
Paediatrics	50	3.64		
Emergency medicine	32	3.6		
Post hoc *analysis* showed a significant difference between obstetrics and gynaecology (highest) and the other nonsurgical specialties.				
**Honour/Integrity**				
Family medicine	53	4.23	2.786	.018
internal medicine	84	4.37		
general surgery	25	4.34		
obstetrics & gynaecology	28	4.56		
paediatrics	50	4.23		
emergency medicine	32	4.2		
Post ho*c analysis* showed a significant difference between obstetrics and gynaecology (highest) and family medicine, paediatrics, and emergency medicine.				

Although the scores differed significantly for altruism and honour/integrity between the residents of obstetrics and other nonsurgical specialties (Table 4), when we categorized the residents into surgical and nonsurgical specialties (Table 5), no significant differences were observed for each domain.

**Table 5.** Comparison of the surgical/nonsurgical specialties in relation to different professionalism domains’ mean scores.

Domains	Mean (SD)	Mean (SD)	*t*-Value	*p*-Value
	Surgical	Nonsurgical		
Respect to others	4.62 (0.38)	4.60 (0.40)	0.38	.707
Excellence/Autonomy	4.38 (0.42)	4.36 (0.39)	0.33	.739
Altruism	3.75 (0.41)	3.64 (0.39)	1.983	.048
Duty/Accountability	4.14 (0.47)	4.08 (0.44)	0.95	.342
Honour/Integrity	4.35 (0.49)	4.30 (0.47)	0.75	.451

#### Analysis of the total professionalism mean scores in relation to residency level, specialty, and gender

Analysis of the mean total scores in relation to two of the demographics variables, namely the year of residency and specialty, did not reveal any statistical significance (Table 6).

**Table 6.** Mean of total professionalism scores in relation to year of residency and specialty.

Variable	Mean	SD	*p*-Value
Residency level			
Junior	4.251	0.029	.678
Senior	4.234	0.029	
Specialty			
Surgery	4.272	0.035	.159
Nonsurgery	4.214	0.022	

*Note*: Gender was not included because it already produced a nonsignificant result for total mean scores, as shown in Table 1.

Similarly, the 2 × 2 × 2 factorial ANOVA of the mean total score in relation to the combination of all three categorical study variables (residency level, specialty, and gender) did not reveal any statistically significant interaction effect (Table 7).

**Table 7.** Factorial analysis of variance to compare the mean total scores in relation to the combination of categorical study variables (gender, specialty, and residency level).

	Mean square	F value	*p*-Value
Residency level * Specialty	0.024	0.263	.609
Specialty * gender	0.128	1.387	.240
Residency level * gender	0.040	0.432	.512
Residency level * Specialty * gender	0.004	0.045	.833

## Discussion

The overall residents’ self-reported attitude towards professionalism was positive. It is notable that there was no significant relationship between either the residents’ level (year) of training or specialty and their attitude towards professionalism. Although not significant, the results show that the overall professionalism scores of male residents were higher than those of female residents. These findings are unique in that they are not in line with the literature.

### High score for respect domain and low for altruism domain

The finding of a high score for respect in the current study is in line with a study by Ojuka [32] but contrasts a study among residents in Iran, which showed the lowest score for respect [33]. Like all religions, Muslims have one faith but different levels of commitment. According to a worldwide survey by the Pew Research Centre’s Forum on Religion & Public Life, these commitment differences can significantly influence and be reflected on the level of orthodoxy, openness, and acceptance of various aspects of Islam [34].

This finding is most likely a reflection of Arabian culture, in which professionalism is a cultural construct [32]. In Arab countries, the cultural constructs/religious teachings enforce “equality” and “respect for all,” particularly the weak and old people. The trust in religious and cultural teaching may have further provided a path for residents to carry out better conduct [35].

A lower score for the item “gives priority to some patients based on social status or nationality” (a negative statement that was reverse coded) is reassuring; this reflects the residents’ belief in equitable access to healthcare services without prejudice based on social status or nationality.

Although altruism is one of the basic tenets of medical professionalism, it scored the lowest compared with the other domains. This finding is in line with. Jauregui [36] who found lower scores. Furthermore, having the altruism domain being lower than that reported in another study among medical students from a comparable Arabian background [30] is a cause for concern. It is important to bear in mind that physician altruism is a complex concept with widely varying interpretations, not an observable behaviour or dichotomous construct that can be readily measured [37]. A study at the University of Leeds showed variability in medical students’ definitions of altruism, who conceptualized it as a spectrum [38]. Altruism, as conceptualized by self-sacrifice, is not always possible or desirable. As suggested by Bishop and Rees, the concept should focus on prosocial behaviour, that is, the balancing of self-interest such as self-care and the interests of others [37]. It is likely that our residents’ responses reflect a reconceptualization of the traditional concept of altruism.

### The residents’ training year

The findings of the current study demonstrate that the total mean score did not have a significant association with the residents’ training year. These findings are in line with Ruiz et al.’s study [39], which concluded that attitude scores at the cognitive and fundamental levels remain fairly unchanged. However, the results are not in line with Woloschuck et al. [14], who noted a persistent decline in several attitude scores as students progressed through their medical education program. Some studies have shown an improvement in attitude scores as learners progress in their program [40].

It is possible that this tool did not pick up on the small changes in attitude and, hence, could not discriminate between levels of training/experience.

Another possible reason for not detecting a difference could be the ceiling effect in the professionalism scores because junior residents possessed acceptable professionalism attitudes, which did not improve significantly during their training program. Other possible explanations include the learning climate, which includes the trainers’ professional behaviour and attitude and who are then seen as role models, and the socialization process within different programs, which may shape both personal and professional values [40]. Specialty training culture, hidden curriculum practice characteristics (workload, work content, patient characteristics, etc.), and societal/cultural factors [41] may also be possible contributors to this finding. Examples of personal factors include residents’ well-being, motivation, and compassion.

Measuring the professionalism scores of faculty members to whom residents are exposed and comparing faculty scores to those of residents in the same specialty may help confirm this hidden curriculum theory. However, a longitudinal study of more than one institute with more years would be necessary to check whether doctors show a rise or decline in their professional attitudes as they progress in their training years. Future studies using qualitative methods across specialties are needed to assess medical professionalism among trainees in more depth.

Professionalism training in postgraduate curricula needs to be better planned [42,43]. The absence of a planned program in the current study setting may be the reason for the nonsignificant differences in professionalism scores over the residents’ training years. This shows the need for a professionalism course in all residency programs. Further support for the introduction of such a program comes from a large surgical residency study in New York University that introduced a professionalism curriculum [44] consisting of an annual, year-long professionalism course to its surgical residency program.

It is important for medical educators to design curricula with an understanding of each generation’s cultural and regional values so that curricula can be successfully aligned and implemented. The literature has also indicated that there are differences among residents in their perceptions of professional attitudes [36,45]. When teaching professional behaviour to residents, it could be useful to address these potential differences.

### The residents’ specialty

Overall, although the mean scores for professionalism were almost similar for all specialties, the altruism and honour/integrity domains were higher among obstetrics and genecology residents than among residents in other specialties. Various factors can be considered here, and it cannot be concluded that a lower score among other specialties means worse professional behaviour.

The literature review carried out for the current study indicated that obstetrics and gynaecology and surgery residents exhibit similar personality characteristics, such as displaying higher extroversion and sensing; these characteristics are known to influence altruism because of the desire they generate to engage with other individuals [46]. This raises the question of whether these findings reflect unique training experiences and work practices.

Our study did not show a significant association between the attitudes of surgical and nonsurgical residents’ professionalism attitude. International studies have found a mixed pattern here. A study by Mianehsaz et al. reported that the mean score of professionalism among residents in surgical specialties was lower than that of nonsurgical specialties [33]. In contrast, Symons et al. reported higher scores among surgical residents than nonsurgical specialties [18].

For practical implications of the results of the current study, program directors should take note that professionalism learning and teaching in postgraduate training needs to be an integral part of the curriculum. This has been highlighted in two studies that found deficiencies in the attitude towards professionalism [42,43], here concluding that residency programs should include well-planned teaching that focuses on professionalism issues relevant to those that residents face in their daily work [44].

Does this mean that there are no specialty-level differences in the training microculture in the Arabian context?

It is not possible to draw firm conclusions that there are no specialty-level differences in the training microculture in the Arabian context because of the limited external validity power of data from a single institute and because of a lack of comparative research; however, it is worth further exploration.

### The residents’ gender

Regarding the influence of residents’ gender, the current study did not find significant differences in professional attitude based on the residents’ gender. This contrasts the findings of a study among Canadian medical students and residents in which females had higher attitude scores than their male counterparts across almost all domains [16]. Similarly, other studies have indicated higher mean attitudinal scores for females [14,15].

Although there was no significant difference between men and women regarding overall professionalism, it is notable that men scored higher in the duty accountability domain. This could be explained by the cultural roles of the two genders in Saudi Arabia, where men have greater social responsibilities and tasks to achieve at home and outside.

## Conclusions

One of the most important findings of the current study is the high score for respect, which could be explained by cultural factors. The lowest mean score was for altruism, which could be because of the residents’ reconceptualization of the traditional concept of altruism. Another notable finding contrary to our expectations is that the overall professionalism score did not improve as the resident received more training. These findings highlight the need for future studies in a wider context. The authors recommend exploring the reasons for these findings by conducting a professionalism survey using a cohort design with trainers and trainees from more than one centre and following up on them on an annual basis. Furthermore, qualitative studies that can elucidate factors that could find explanations, such as the trainers’ attitude, the hidden curriculum, and the learning environment.

## Limitations

The current study has several limitations, as follows:

- First, the present study was a self-perception online survey that provided a snapshot on residents’ attitude towards professionalism at one point in time. Self-perception at one point in time may not be the best measure of attitude.
- Second, because “professionalism” is an umbrella term used to include abilities/attributes such as “communication, teamwork, ethics, attitudes, and so forth,” some of the items of the LAMPS instrument may address these abilities/attributes individually, rather than collectively, which may introduce some bias.
- Third, the current study involved participants from one institute. The results should not be unduly generalised to other institutes. Even though this institute is one of the largest in the Arab world and the study residents graduated from different medical schools all over the kingdom, one needs to be cautious in generalising the results.

## Strengths

The following strengths seem to outweigh these limitations:

- The present study has added to the growing literature on resident perspectives on professionalism and can be seen as an initial study combining the effects of several demographic variables within the same study.
- The study was conducted in a large medical city and involved a representative sample of residents from different specialties; in addition, the residents graduated from medical schools that are in different parts of the country.
- It is also one of the first few studies that attempted to explore the views and attitudes of residents, who will be the future heads of the health system.
- The selected tool was originally developed for the Arabian context, which is in contrast to other tools developed in other contexts.

### Summary

Contrary to the findings in the literature, no significant differences were observed in the overall attitudes towards professionalism among the residents regarding training year, gender, and specialty. There may be numerous reasons for these findings, such as the effect of the study design and study tool. The effect of sociocultural factors on the variation in attitudes towards professionalism, however, cannot be ruled out. The low altruism score and the absence of an improvement of the total score as the resident gained more experience and training were found to be areas that need further investigation. Therefore, we suggest that future research look for possible explanations using multi-institutional surveys to explore not only the residents’ attitudes, but also the trainers’ attitudes and practices, work situations, hidden curriculum, and culture.

## Data Availability

The data that support the findings of the current study are available from the corresponding author upon reasonable request.

## Appendix

### Survey instrument

Demographics

**Table A1.** The 31-item modified LAMPS domains and the means (SD) (*n* = 272).

Behavioural item within domains “Do you agree when the doctor … ?”	Positive statement	Mean	SD
Duty/Accountability			
1. Encourages patients to contribute to decision making	Yes	4.53	0.625
2. Discusses patients’ cases with colleagues in a crowded elevator	No	4.24	0.846
3. Calls the insurance company to follow up on a valid patient claim	Yes	3.12	1.199
4. Actively participates in orientation for new residents	Yes	4.58	0.576
5. Leaves before handing over patients to the next colleague on duty	No	4.68	0.664
6. Admits a wrong diagnosis before a patient	Yes	3.71	1.249
7. Declines an invitation to an infection control committee meeting	No	3.80	0.916
Excellence/Autonomy			
1. Searches for the best evidence available in patient care	Yes	4.73	0.444
2. Reflects on clinical cases to discover his/her unmet learning needs	Yes	4.57	0.678
3. Makes a deal with a pharma company to sponsor his/her conference	No	4.31	0.829
4. Collaborates with colleagues to draft new hospital guidelines	Yes	4.42	00.69
5. Attends patient’s questions to explain their illness in a busy clinic	Yes	4.54	0.575
6. Invests part of his/her income on attending medical conferences	Yes	3.63	0.951
**Altruism**			
1. Frequently skips clinical teaching to prepare for a conference	No	3.78	0.762
2. Cancels a family appointment for an urgent patient’s need	Yes	3.53	0.909
3. Stands in as a witness against the employer hospital in favour of a patient before the court	Yes	3.60	0.685
4. Turns down a home visit to a disabled patient because of a busy clinic	No	3.14	0.809
5. Declines going to a sports club to respond to an emergency call	Yes	4.31	0.660
***Honour/Integrity***			
1. Issues a false sick leave for a kid of a friend to study home	No	4.45	0.686
2. Introduces medical students as doctors to patients	No	3.89	1.060
3. Hides information about fatal diagnosis to avoid patient disturbance	No	3.92	1.051
4. Gives wrong information to a patient to protect a colleague	No	4.54	0.562
5. Changes actual data in his/her research based on supervisor’s advice	No	4.55	0.752
6. Signs the attendance paper in scientific meetings on behalf of his colleagues and asks them to sign it in his/her absence	No	4.15	0.952
7. Take the work or idea from a colleague and passes it off as one’s own without acknowledging it or purchasing work from a supplier	No	4.71	0.455
Respect			
1. Respects the roles of all members of the healthcare team in the department	Yes	4.69	0.714
2. Keeps patients waiting in his/her clinic without apology	No	4.65	0.537
3. Gives priority to some patients based on social status or nationality	No	4.74	0.503
4. Considers patient background when explaining their clinical illness	Yes	4.45	0.776
5. Criticises a prescription written by a colleague in front of patients	No	4.47	0.718
6. Is late for scientific meetings with colleagues and medical staff without excuse	No	4.66	0.475

**Table:**

Gender:	Male			Female		
Year of residency:	R1	R2	R3	R4	R5	
Specialty:	Internal Medicine	Family Medicine	Paediatrics	Emergency Medicine	Obstetrics and Gynaecology	General Surgery

**Table:**

Domains	Strongly Agree	Agree	Natural	Disagree	Strongly Disagree
Duty/Accountability					
Encourages patients to contribute to decision making					
Discusses patients’ cases with colleagues in a crowded elevator					
Calls the insurance company to follow up on a valid patient claim					
Actively participates in orientation for new residents					
Leaves before handing over patients to the next colleague on duty					
Admits a wrong diagnosis before a patient					
Declines an invitation to an infection control committee meeting					
Excellence/Autonomy					
Searches for the best evidence available in patient care					
Reflects on clinical cases to discover his/her unmet learning needs					
Makes a deal with a pharma company to sponsor his/her conference					
Collaborates with colleagues to draft new hospital guidelines					
Attends to patient’s questions to explain their illness in a busy clinic					
Invests part of his/her income on attending medical conferences					
Altruism					
Frequently skips clinical teaching to prepare for a conference					
Cancels a family appointment for an urgent patient’s need					
Stands in as a witness against the employer hospital in favour of a patient before the court					
Turns down a home visit to a disabled patient because of a busy clinic					
Declines going to a sports club to respond to an emergency call					
Honour/Integrity					
Issues a false sick leave for a kid of a friend to study home					
Introduces medical students as doctors to patients					
Hides information about a fatal diagnosis to avoid patient disturbance					
Gives wrong information to a patient to protect a colleague					
Changes actual data in his/her research based on supervisor’s advice					
Signs the attendance paper in scientific meetings on behalf of his/her colleagues and asks them to sign it in his absence					
Takes the work or idea from a colleague and passes it off as one’s own without acknowledging it or purchasing work from a supplier					
Respect					
Respects the roles of all members of the healthcare team in the department					
Keeps patients waiting in his/her clinic without apology					
Gives priority to some patients based on their social status or nationality					
Considers patient background when explaining their clinical illness					
Criticises a prescription written by a colleague in front of patients					
Is late for scientific meetings with colleagues and medical staff without excuse					

## Abbreviations

LAMPS: Learners Attitude of Medical Professionalism Scale
ABIM: American Board of Internal Medicine
KSUMC: King Saud University Medical City
